# Different effects of visual occlusion on interpersonal coordination of head and body movements during dyadic conversations

**DOI:** 10.3389/fpsyg.2024.1296521

**Published:** 2024-08-02

**Authors:** Kentaro Kodama, Daichi Shimizu, Ken Fujiwara

**Affiliations:** ^1^University Education Center, Tokyo Metropolitan University, Tokyo, Japan; ^2^Graduate School of Human Development and Environment, Kobe University, Kobe, Japan; ^3^Department of Psychology, National Chung Cheng University, Chiayi, Taiwan

**Keywords:** interpersonal coordination, synchrony, perceptual coupling, visual information, compensatory behavior, cross-recurrence quantification analysis

## Abstract

**Introduction:**

In recent decades, interpersonal coordination and synchrony have been extensively examined in the field of psychology and cognitive science. Studies suggest that perceptual information enables interpersonal coordination and that perceptual noise may even enhance coordination. However, how these perceptual factors influence interpersonal coordination dynamics between head and body movements remains unclear. This study investigated the effect of visual information on the interpersonal coordination of head and body movements during dyadic conversations.

**Methods:**

The availability of visual information was manipulated by positioning a partition halfway between a pair of participants, and the conversations were recorded using a video camera. A video-based human pose estimation software (OpenPose) was used to quantify each interlocutor’s head and body movements, which were submitted for cross-recurrence quantification analysis (CRQA), to assess the degree of coordination between the interlocutors.

**Results:**

The results showed different effects between head- and body-movement coordination (i.e., a CRQA measure, *maximum line length*). The occlusion of visual information increased head-movement coordination, whereas it decreased body-movement coordination.

**Discussion:**

The results suggest that a distinct mechanism may be present at the head- and body-movement coordination level and this study observed differing appearances of compensatory behaviors. Further studies should be conducted to investigate the complex relationships between interpersonal coordination dynamics and various kinds of communication constraints, such as long-term or short-term, and lower-order (perceptual-motor) or higher-order (cognitive-social) level constraints.

## 1 Introduction

Recently, the concept of embodiment has received significant attention in psychology and cognitive science (e.g., Chemero, 2011). Traditional and classical psychology supposes that the mind is independent of the body. However, from the aspect of embodiment, recent studies have demonstrated both theoretically and empirically that we cannot separate the mind from the body and its environment including others and its context (e.g., Anderson et al., 2012; Riley et al., 2012). Cognitive processes and motor action and behavior are interdependent and interact with each other.

Moreover, cognitive processes involving social factors, such as language and communication, are supposedly embodied (e.g., Richardson et al., 2008; Shockley et al., 2009). For example, nonverbal behaviors (such as gestures) are believed to reflect cognitive processes (e.g., Streeck, 2015). Movement coordination and synchrony between participants have been observed and described in early social interaction studies (e.g., Kendon, 1970). Therefore, interpersonal bodily coordination is considered to be essential for social cognition and interaction (e.g., Knott, 2012; Tschacher and Bergomi, 2015). Such bodily coordination and resonance are also said to embody affective factors such as empathic understanding (Fuchs, 2017).

In line with this approach, the mind and cognition could be considered as a complex phenomenon emerging from the body-environment interaction, which can be regarded as a self-organizing phenomenon. In particular, based on the dynamical systems or self-organization theory, these approaches are referred to as dynamical systems approaches and have been applied to interpersonal coordination and dyadic interaction studies (e.g., Schmidt et al., 2011; Dale et al., 2013; Kyselo and Tschacher, 2014).

Interpersonal coordination and synchrony have been extensively examined across a broad continuum, from perceptual-motor *low-level* processes (e.g., Tognoli et al., 2007; Schmidt et al., 2011) to cognitive-social *high-level* processes (e.g., Garrod and Pickering, 2009; Paxton and Dale, 2013). Bodily coordination between interlocutors, such as postural and head movement coordination, can change depending on linguistic factors (Shockley et al., 2007), communication type (Paxton and Dale, 2013), and social relationships (Fujiwara et al., 2020) during verbal communication. Conversely, it also affects sociopsychological factors such as affiliation and likability between interlocutors (Hove and Risen, 2009). However, research on the low-level constraints of interpersonal coordination, such as perceptual information, is limited (Paxton and Dale, 2017).

Our movements are coordinated with those of other people during conversations, even without visual information (Shockley et al., 2003). In other words, interpersonal coordination can emerge through verbal interactions that use only auditory information. A previous study found a significant increase in interpersonal coordination (e.g., head-movement coordination) between participants in the presence of auditory noise (Boker et al., 2002). The researchers interpreted that the participants coupled their movements more closely with each other when verbal communication became more difficult. Recently, increased synchrony (i.e., movement coherence) in communication when background noise conditions are more difficult has been reported (Hadley and Ward, 2021). These studies show that auditory information, such as background noise, could affect and enhance interpersonal coordination among participants. Additionally, visual noise is assumed to increase interpersonal coordination (Paxton and Dale, 2017). Paxton and Dale (2017) manipulated visual stimuli by asking participants to wear special glasses and adapt flashing screens on glass. They hypothesized that changing visual information interpreted as noise increases head-movement coordination, which partially increases depending on the conversational context. These findings suggest that perceptual information enables the coordination of body movements with other people. They also indicate that perceptual noise, which complicates communication, may enhance bodily coordination.

The notion that perceptual noise can boost interpersonal coordination can be interpreted as compensatory behavior from the perspective of *interpersonal synergy*, defined as higher-order control systems formed by coupling the degrees of freedom in the movement systems of two (or more) actors (Riley et al., 2011). *Reciprocal compensation* is among the characteristics of synergies and refers to the ability of one component of the synergy to react to changes in another component (Riley et al., 2011). Black et al. examined this compensatory behavior in interpersonal rhythmic motor coordination (Black et al., 2007). They found the presence of synergies for interpersonal coordination at the lower-order perceptual-motor level and argued that synergies are not hard-wired features of an actor’s neuromuscular system. Instead, they are emergent properties of perception–action systems linked together informationally (e.g., visually; Black et al., 2007; Riley et al., 2011). Duran and Fusaroli addressed interpersonal coordination in deception and disagreement situations (Duran and Fusaroli, 2017). They reported that deceptive conversations showed increased head movement coordination with a peak in deceptive disagreement conversations. Their results suggest that higher-order communicative constraints (e.g., deception and conflict) can shape low-level interpersonal coordination (e.g., head-movement coordination), which can be described as specific modalities of multimodal interpersonal synergy. The findings of these previous studies might suggest that one component of synergy (e.g., perceptual modality) can react and adapt to changes in other components at various communication levels, including changes of the different body parts (e.g., head and body). From the viewpoint of interpersonal synergy, perceptual noise or the unavailability of perceptual information might induce compensatory behavior and result in increased interpersonal coordination.

Ramseyer and Tschacher (2014) investigated interpersonal synchrony between patients and therapists in psychotherapy. They separately quantified head and body movement synchrony, and assessed both micro-outcomes using self-reported post-session questionnaires and macro-outcomes via questionnaires that quantified the attainment of treatment goals as well as changes in experience and behavior at the end of therapy (Ramseyer and Tschacher, 2014). Their results indicated that head synchrony predicted the global outcome of therapy and body synchrony predicted session outcomes. They argued that the separation of head and body synchrony suggests that distinct mechanisms may operate in these two regions: that head synchrony embodied phenomena along with temporal extension (overall therapy success), whereas body synchrony embodied phenomena of a more immediate nature (session-level success; Ramseyer and Tschacher, 2014). The differences between head and body movements are not clear; however, their functions might differ between interlocutors during conversational processes. For example, speakers move their heads when they talk in association with their utterances. Similarly, listeners also move their heads to show their understanding and agreement (i.e., nodding; Hale et al., 2020). In contrast, speakers move their upper body, excluding the head, particularly when making hand gestures. However, listeners might not move their upper body as often as speakers do. Considering these functional differences in head and body movements between speakers and listeners, as well as previous findings of psychotherapy studies (Ramseyer and Tschacher, 2014), different dynamics between head and body movements can be expected depending on the availability of perceptual information.

This study examined the effect of visual information on the interpersonal coordination of head and body movements during dyadic conversations. Previous studies on the effect of perceptual information and noise suggest that interpersonal coordination may enhance communication signals in a noisy environment (Paxton and Dale, 2017). Accordingly, this study hypothesized that the unavailability of visual information may increase interpersonal bodily coordination. Perceptual noise and the unavailability of perceptual information may seem distinct; however, they can be assumed to impact perceptual systems in a similar manner when viewed from the perspective of reciprocal compensation within the notion of interpersonal synergy. Reciprocal compensation, as observed in compensatory behavior, refers to the ability of one component of synergy to respond to changes in the other components (Riley et al., 2011). Both perceptual noise and the unavailability of perceptual information can represent alterations for perceptual systems; therefore, synergy is expected to react and adapt to these changes to accomplish tasks, such as communication with others. A distinct mechanism of head and body synchrony has been posited (Ramseyer and Tschacher, 2014). Therefore, we explored whether head- and body-movement coordination could display differing dynamics.

## 2 Materials and methods

### 2.1 Participants

A total of 52 pairs of participants (17 female pairs; age (mean ± SD), 20.06 ± 1.15 years; all native Japanese speakers) were recruited. All participants knew each other before the experiment and recognized themselves as friends (*M*_duration_ = 28.85 months, *SD*_duration_ = 35.54 months).

### 2.2 Apparatus

A video camera (HDR-PJ800, SONY) was placed in front of the participants at a distance of 280 cm, and it was used to record their body movements (frame rate was 30 FPS). MATLAB (R2020b, MathWorks) and RStudio (1.4.1103) were used to analyze the data.

### 2.3 Procedure

**Figure 1:**
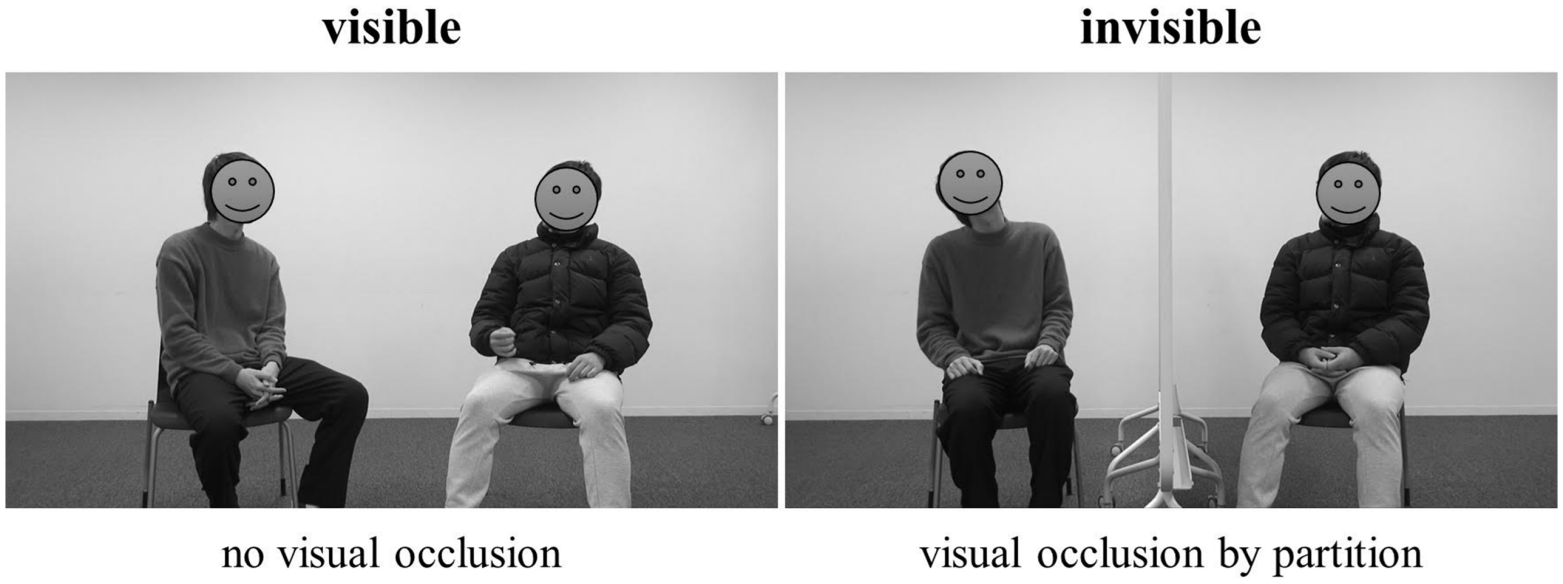
Experimental situation. (Left: visible condition; Right: invisible condition).

Two conditions were compared: the visible condition, in which both visual and auditory information were available, as in the natural situation shown in Figure 1 left; and the invisible condition, in which only auditory information was available as a partition was positioned halfway between the two participants (i.e., a within-subject design). In the invisible condition, the participants could not see each other’s gestures. Participants were instructed to have 6-min conversations to talk, get to know each other better, and deepen their relationship. As the conversation topics were not specified, most participants talked about each other’s recent activities. The pairs underwent each condition; the order of the two conditions was counterbalanced across pairs of participants.

### 2.4 Data analysis

An automated objective video analysis algorithm was performed in Ubuntu 18.04 on a laptop computer (XPS7390, DELL) with OpenPose version 1.5.1 to quantify the extent to which each participant moved (OpenPose, Cao et al., 2017). It estimated the two-dimensional coordinate information of the joint body parts. Fifteen coordinate points (including the nose, eyes, neck, shoulders, elbows, wrists, left and right hips, mid-hip, and knees) were used for the analysis. The ankles were excluded from the analysis because they were frequently out of the frame. To compensate for the missing values of the coordinates, linear interpolation was performed using the *filloutlier* function of MATLAB. The distance of each coordinate between frames was calculated using the Pythagorean theorem to obtain the movement time series. The distances of the nose and eyes were summed for head movements, and the other 12 distances were summed to represent body movements, which occurred throughout the conversation.

To quantify the degree of interpersonal coordination between the participants, a nonlinear time series analysis, referred to as *cross-recurrence quantification analysis* (CRQA),^1^ was applied between two time series of each participant’s head and body movements (Figure 1). CRQA captures a dynamic system’s recurring properties and patterns resulting from two streams of information interacting over time (Zbilut et al., 1998). RQA was originally developed to uncover subtle time correlations and repetitions of patterns. Moreover, it is relatively free of assumptions about data size and distribution (Zbilut and Webber, 1992). In CRQA, two time-delayed copies of the original time series are used to embed the data in a higher-dimensional space to further analyze the recurrent structure between them (Zbilut et al., 1998).

This study calculated two CRQA measures, namely, *percentage of recurrence* (%REC) and *maximum line length* (MAXL). For interpersonal coordination, %REC in CRQA corresponds to the ratio of the actual number of shared locations to the number of possible shared locations in the phase space (Shockley, 2005). This implies that a higher %REC indicates less noise in the system. In other words, it indicates that the system is more stable. The other measurement is related to the line structure calculated from the recurrence plot (MAXL). This is the longest shared trajectory in the phase space and the length of the maximum diagonal line on the plot (Webber and Zbilut, 2005). MAXL is a measure of the stability of a shared activity (Shockley, 2005) and provides an index of the system’s sensitivity to perturbations (i.e., the strength of the attractor against perturbations; Pellecchia et al., 2005).

After filtering raw time series data using the Savitzky–Golay filter (order 3, length 11), the optimal values for the input parameters were determined, with reference to the standard guidelines for the RQA method (Webber and Zbilut, 2005). CRQA was performed using the MATLAB toolbox Cross-Recurrence Plot Toolbox version 5.21 (Marwan and Kurths, 2002). Consequently, parameters of 30 were chosen for time delay, 7 for embedding dimensions, and 0.6 for the radius within the Euclidean norm between normalized vectors. A generalized linear mixed model^2^ analysis was also performed using the R package glmmML (RStudio Team). The datasets comprised the CRQA measures (%REC and MAXL of head and body) as dependent variables, condition (including visible or invisible) as an independent variable, and duration (relationship period) as a control variable because it can affect the degree of synchrony (Fujiwara et al., 2020); pair (*N* = 52) was included as a random variable.^3^

## 3 Results

Table 1 shows the mean and standard deviation (SD) values of %REC and MAXL for each condition. For head-movement coordination, the %REC was 2.33% (SD = 0.79) and 3.13% (SD = 1.50) and MAXL was 65.85 (SD = 59.71) and 95.90 (SD = 70.98) in the visible and invisible conditions, respectively. For body-movement coordination, the %REC was 5.36% (SD = 3.53) and 3.13% (SD = 5.73) and MAXL was 278.00 (SD = 135.40) and 272.15 (SD = 185.53) in the visible and invisible conditions, respectively.

**Table 1:** Mean and standard deviation (SD) of cross-recurrence quantification analysis (CRQA) measures (%REC: percentage of recurrence, MAXL: maximum line length) for each condition and each body part.

Body part	CRQA	Visible		Invisible	
		Mean	SD	Mean	SD
Head	%REC	2.33	0.79	3.13	1.50
	MAXL	65.85	59.71	95.90	70.98
Body	%REC	5.36	3.53	6.30	5.73
	MAXL	278.00	135.40	272.15	185.53

**Figure 2:**
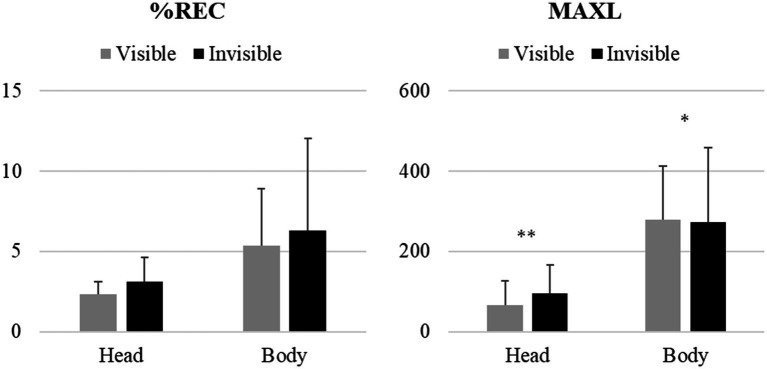
Means and Standard Deviations (SD) of the Cross-Recurrence Quantification Analysis (CRQA) measurements (%REC, percentage of recurrence; MAXL, maximum line length) for each condition and body part (Left: visible condition; Right: invisible condition). ^*^p < 0.05, ^**^ *p* < 0.001.

Table 2 presents the results of the generalized linear mixed model for the head- and body-movement coordination parameters (i.e., %REC and MAXL). No significant effect of condition in the %REC of the head- or body-movement coordination (*p* = 0.727 and *p* = 0.773, respectively) was observed. However, a significant positive effect of head-movement coordination (*p* < 0.001) as well as a significant negative effect of body-movement coordination (*p* < 0.05) in MAXL were noted (Figure 2).

**Table 2:** Results of the generalized linear mixed model for cross-recurrence quantification analysis (CRQA) Measures (%REC: Percentage of Recurrence, MAXL: Maximum Line Length) for each body part.

Body part	CRQA		coef	*SE* (coef)	*z*	*p* (>|z|)
Head	%REC	(Intercept)	−3.771	0.731	−5.156	0.000
		condition	0.301	0.860	0.350	0.727
		duration	0.001	0.011	0.113	0.910
	MAXL	(Intercept)	3.967	0.120	32.985	0.000
		condition	0.376	0.016	23.962	0.000^**^
		duration	0.000	0.003	0.162	0.871
Body	%REC	(Intercept)	−2.848	0.499	−5.707	0.000
		condition	0.171	0.594	0.288	0.773
		duration	−0.000	0.009	−0.090	0.928
	MAXL	(Intercept)	5.506	0.093	59.384	0.000
		condition	−0.021	0.008	−2.542	0.011^*^
		duration	0.000	0.002	0.003	0.998

* *p* < 0.05.

** *p* < 0.01.

## 4 Discussion

In this study, we examined how visual information affected the interpersonal head and body movement coordination during dyadic conversations. We hypothesized that the unavailability of visual information might increase interpersonal bodily coordination. Furthermore, we explored whether head and body movement coordination could display differing dynamics. We quantified and assessed the degree of interpersonal coordination using %REC (coordination stability) and MAXL (coupling strength) according to previous studies (e.g., Shockley, 2005).

Although the difference in %REC was insignificant, the generalized linear mixed model revealed different effects in MAXL between head- and body-movement coordination. Regarding head-movement coordination, a positive effect suggested that the coupling strength (i.e., MAXL) increased in the invisible condition. However, regarding body-movement coordination, a negative effect suggested that the coupling strength decreased in the invisible condition. Thus, the current hypothesis—that the unavailability of visual information may increase interpersonal bodily coordination—was partly supported. Specifically, the occlusion of visual information increased head-movement coordination, but decreased body-movement coordination. Thus, the occlusion of visual information affected head- and body-movement coordination differently, as we predicted. This finding is interesting, but the question of how these complex results can be interpreted arises.

### 4.1 Different effects in head- and body-movement coordination

Generally, head movements (e.g., nodding) serve various roles and functions, such as indicating agreement or empathy, for both listeners and speakers (e.g., McClave, 2000; Aoki, 2011). Conversely, body movements encompass hand movements (i.e., gestures), which serve different roles and functions, such as substituting for speech or conveying speakers’ unspoken thoughts (e.g., Goldin-Meadow, 1999; McNeill, 2000). Accordingly, head and body movements play different roles in communication. Therefore, interpersonal coordination dynamics can also vary between head and body movements, and their meanings can differ depending on these coordination levels.

For head-movement coordination, the invisible condition (i.e., unavailability of visual information) may enhance or boost the communication signal, as predicted by previous studies (Paxton and Dale, 2017). This result can be interpreted in terms of the relationship between visibility and gesture production. When participants could not see each other, they interacted in a relatively simple way using auditory information only. In such an invisible condition, coupling and or entrainment between the speakers’ head movements with utterances and listeners’ nodding movement in response to the speakers’ voices might occur through auditory verbal interaction (Shockley et al., 2003, 2007). We consider that these interactions throughout the auditory modality might increase head-movement coordination in the invisible condition.

However, for body-movement coordination, a speakers’ gestures decrease when the visibility between the speaker and listener is blocked (i.e., the invisible condition; e.g., Emmorey and Casey, 2001). Consequently, we consider that body movements, including gestures, and their coordination might decrease in the invisible condition. The difference between conditions in terms of the number and type of gestures should be investigated in future studies (Alibali et al., 2001).

### 4.2 Distinct mechanism and functions of head- and body-movement coordination

Previous research argues that distinct mechanisms may operate at these two levels (Ramseyer and Tschacher, 2014). Head-movement coordination embodied phenomena have been associated with temporal extension on a longer-term scale, whereas body-movement coordination embodied phenomena have a more immediate nature on a short-term scale (Ramseyer and Tschacher, 2014). Given these distinct mechanisms, the findings of the current study suggest that visual information during conversations could influence long-term phenomena, as reflected in head-movement coordination. However, the study by Ramseyer and Tschacher used the term long-term to refer to global therapy success (i.e., a macro-outcome; Ramseyer and Tschacher, 2014). This timescale typically spans several weeks, months, or even years. The present study could not address long-term phenomena within such an extended temporal scale. Thus, changes in the relationship between bodily coordination (i.e., at the head- or body-movement level) and embodied social and cognitive phenomena (e.g., long- or short-term) may be possible, depending on the communication type and available perceptual information.

Particularly in psychotherapy, the head movement of nodding plays an important role among therapists in displaying their understanding of, alignment with, and empathy toward clients (Muntigl et al., 2012; Graf et al., 2014). This nonverbal behavior can be used as a clinical technique in psychotherapy that enables rapport building between the therapist and client and has a long-term influence on the therapist–client relationship. Therefore, head-movement coordination (i.e., nodding) can relate to long-term aspects of a specific communication type (e.g., psychotherapy).

In natural conversations, the head movement of nodding does not always have the same function it has in psychotherapy and may have various functions (McClave, 2000). When participants have already built social relationships as friends, as they had in this experiment, they may not intend to expressly display their understanding, alignment, and empathy. Additionally, when participants cannot see each other, nodding may have different functions, such as enhancing the communication signal through auditory information (Paxton and Dale, 2017), after which head-movement coordination can increase. In such cases, interpersonal coordination can be organized through perceptual coupling via auditory information (Shockley et al., 2003), which can be regarded as a fast-changing phenomenon on a short-term scale (Dale, 2015). Therefore, head-movement coordination may not always embody long-term and or slow-changing phenomena.

The results suggest that a distinct mechanism may exist at the head- and body-movement coordination level. We also speculate that the relationship between coordination dynamics at each level (i.e., head or body) and embodied social and cognitive phenomena (e.g., long- or short-term aspects) may change depending on the communication type and available perceptual information. Further investigations to clarify this hypothesis are needed.

### 4.3 Unavailability of perceptual information and its compensation

Activation of the auditory mirror neuron system during the perception of sounds and speech have been observed (e.g., Gazzola et al., 2006). Thus, participants in the current experiment may have synchronized and coordinated their body movements even without visual information. However, from the viewpoint of interpersonal synergy, the mirror neuron system model is not expected to explain reciprocal compensation (Riley et al., 2011), which involves compensation among various components such as individuals and multimodality.

As mentioned in the introduction, the results can also be discussed in terms of compensatory behavior from the perspective of interpersonal synergy (Riley et al., 2011). Reciprocal compensation is among the characteristics of synergies and the ability of one component of synergy to react to changes in other people (Riley et al., 2011). Previous studies on compensatory behavior in interpersonal synergy suggest that one component of interpersonal synergy (e.g., an individual or a modality) can react and adapt to changes in other components at various communication levels in complicated ways. The results might show differing appearances of compensatory behaviors in different body parts (i.e., head or body) in adapting to visual occlusion during conversation. Previous studies on compensatory behavior during communication examine only one aspect of behaviors (e.g., head movement). However, this study compared different aspects, both head and body movements, and observed differing appearances of compensatory behaviors. Further experimental studies should be conducted to ascertain the complex relationships between interpersonal coordination dynamics and various kinds of communication constraints, such as long-term (slow changing) or short-term (fast changing) and lower-order (perceptual-motor) level or higher-order (cognitive-social) level.

### 4.4 Limitations

First, the present study had theoretical limitations. As discussed above, further investigation is required to experimentally reveal the complex interactions and compensations among multilevel components in the future. Additionally, debate on how we can theoretically explain the complex relationships observed in the current data is needed. Second, this study had technical limitations. OpenPose shares the same limitation regarding the two- versus three-dimensional problem as the frame differencing method, which utilizes only one camera (e.g., *Motion Energy Analysis* used in Ramseyer and Tschacher, 2014).

## Data availability statement

The raw data supporting the conclusions of this article will be made available by the authors, without undue reservation.

## Ethics statement

The studies involving humans were approved by The research ethics committee of the Osaka University of Economics. The studies were conducted in accordance with the local legislation and institutional requirements. Written informed consent for participation in this study was provided by the participants’ legal guardians/next of kin.

## Author contributions

KK: Writing – review & editing, Writing – original draft, Visualization, Validation, Supervision, Software, Resources, Project administration, Methodology, Investigation, Formal Analysis, Data curation, Conceptualization. DS: Writing – review & editing, Methodology, Formal Analysis, Conceptualization. KF: Writing – review & editing, Validation, Supervision, Resources, Project administration, Methodology, Investigation, Funding acquisition, Formal Analysis, Data curation, Conceptualization.

## Footnotes

^1^Cross-recurrence quantification analysis (CRQA) is a nonlinear time-series analysis developed to capture the dynamics of complex systems. According to a previous study (Richardson and Chemero, 2014), complex dynamical systems possess three characteristics: (1) They are composed of numerous components or agents; (2) They show emergent behavior wherein their collective behavior exhibits a coherent pattern that cannot be predicted from the behavior of the individual components; and (3) The emergent behavior is self-organized, indicating that it does not result from a controlling individual component or agent. Complex system theory and nonlinear dynamics have provided not only theoretical concepts and models but also analytical tools. To capture and quantify the dynamics of complex systems, conducting recurrence analysis (e.g., CRQA) for behavioral data involves plotting the recurrent points on a two-dimensional plot (i.e., a recurrence plot) based on whether recorded points, states, or events in a time series are revisited or reoccur over time (Richardson and Chemero, 2014). This plot provides a visualization of the revisiting or recurring patterns in a system’s behavioral state space, which can then be quantified using (Cross-)RQA to identify the underlying structure of the dynamics (Richardson and Chemero, 2014). Representative RQA measures, %REC, and MAXL, are calculated as: ﻿﻿￼﻿, ﻿﻿￼﻿ respectively (see Marwan et al., 2007 for detailed descriptions).

^2^A binomial distribution for the %REC and a Poisson distribution for MAXL was assumed.

^3^To reveal the adequacy of our sample size (N = 52), we conducted a power analysis, performed at a significance level of 5% (*α* = 0.05). The effect sizes were defined according to Cohen’s conventions (*d*). Our power analysis yielded powers of 32.7, 84.8, and 98.9% for small (*d* = 0.2), medium (*d* = 0.5), and large (*d* = 0.8) effect sizes, respectively. These results indicate that our study had adequate power to detect medium and large effects.
